# Post-vaccination, post-infection and hybrid immunity against severe cases of COVID-19 and long COVID after infection with SARS-CoV-2 Omicron subvariants, Czechia, December 2021 to August 2023

**DOI:** 10.2807/1560-7917.ES.2024.29.35.2300690

**Published:** 2024-08-29

**Authors:** Martin Šmíd, Tamara Barusová, Jiří Jarkovský, Ondřej Májek, Tomáš Pavlík, Lenka Přibylová, Josefína Weinerová, Milan Zajíček, Jan Trnka

**Affiliations:** 1 Centre for Modelling of Biological and Social Processes, Prague, Czechia; 2 Department of Econometrics, Institute of Information Theory and Automation, Czech Academy of Sciences, Prague, Czechia; 3 Department of Probability and Mathematical Statistics, Faculty of Mathematics and Physics, Charles University, Prague, Czechia; 4 Institute of Biostatistics and Analyses, Ltd., Brno, Czechia; 5 Institute of Biostatistics and Analyses, Faculty of Medicine, Masaryk University, Brno, Czechia; 6 Institute of Health Information and Statistics of the Czech Republic, Prague, Czechia; 7 Department of Mathematics and Statistics, Faculty of Science, Masaryk University, Brno, Czechia; 8 School of Psychology, University of Nottingham, University Park, Nottingham, United Kingdom; 9 Department of Biochemistry, Cell and Molecular Biology, Third Faculty of Medicine, Charles University, Prague, Czechia; * All authors contributed equally to this work.

**Keywords:** covid-19, vaccine effectiveness, hybrid immunity, waning, BA1/2, BA4/5, long covid

## Abstract

**Background:**

COVID-19 remains a major infectious disease with substantial implications for individual and public health including the risk of a post-infection syndrome, long COVID. The continuous changes in dominant variants of SARS-CoV-2 necessitate a careful study of the effect of preventative strategies.

**Aim:**

We aimed to estimate the effectiveness of post-vaccination, post-infection and hybrid immunity against severe cases requiring oxygen support caused by infections with SARS-CoV-2 variants BA1/2 and BA4/5+, and against long COVID in the infected population and their changes over time.

**Methods:**

We used a Cox regression analysis with time-varying covariates and calendar time and logistic regression applied to national-level data from Czechia from December 2021 until August 2023.

**Results:**

Recently boosted vaccination, post-infection and hybrid immunity provide significant protection against a severe course of COVID-19, while unboosted vaccination more than 10 months ago has a negligible protective effect. The post-vaccination immunity against the BA1/2 or BA4/5+ variants, especially based on the original vaccine types, appears to wane rapidly compared with post-infection and hybrid immunity. Once infected, however, previous immunity plays only a small protective role against long COVID.

**Conclusion:**

Vaccination remains an effective preventative measure against a severe course of COVID-19 but its effectiveness wanes over time thus highlighting the importance of booster doses. Once infected, vaccines may have a small protective effect against the development of long COVID.

**Key public health message**

**What did you want to address in this study and why?**

Protective effects of immunity against COVID-19 have been shown to diminish over time at varying speed depending on the source of immunity (infection, vaccination or a combination of both), viral variant, age and other factors. Using national-level data from Czechia, we here wanted to assess the protection afforded by vaccines and/or previous infection against severe COVID requiring oxygen support or long COVID up to 16 August 2023.

**What have we learnt from this study?**

Protection provided by the original vaccination series wanes fast but can be prolonged through the administration of booster doses, especially with the updated bivalent mRNA vaccines. Immunity after infection or hybrid immunity (from infection and vaccination) provided significantly better protection, waning more slowly over time. The protective effect of vaccines on the development of long COVID in the infected population was small.

**What are the implications of your findings for public health?**

Vaccination against COVID-19 remains an effective strategy to prevent a severe course of this illness but recent booster doses are required for a significant protection. Since hybrid immunity provides the strongest protection against severe cases, vaccination can be recommended even to individuals who have recovered from the disease. The protective effect of vaccination against long COVID, once infected, appears small.

## Introduction

With COVID-19 becoming endemic, quantification of protection by various sources of immunity is necessary, be it post-infection, post-vaccination or hybrid immunity. In this study, we focus on two serious outcomes of the disease: severe course needing oxygen support and post-acute COVID-19 syndromes colloquially called long COVID.

Previous work on the immunity against a symptomatic and severe course include controlled clinical trials [1-5], whole-population observational studies [6-10] and meta-analyses [11-13]. Published results show a significant protective effectiveness of primary series vaccinations and booster doses against COVID-19, hospitalisation, severe course and death. A gradual, time-dependent decrease in the effectiveness of mRNA-based vaccines from the point of vaccination and its augmentation after additional doses is well supported in the literature for several variants of severe acute respiratory syndrome coronavirus 2 (SARS-CoV-2) [6-8,10], including studies on the Omicron variant and its sub-variants [9,14-17] and a recent meta-analysis [18]. Fewer effectiveness data are available for more recent mRNA vaccines updated for the Omicron sub-variants, namely Comirnaty bivalent Original/Omicron BA.4–5 mRNA vaccine (BNT162b2 mRNA, BioNTech-Pfizer), outside of clinical trials [8,19].

The relationship between vaccination and long COVID is less well studied. Vaccines can decrease the risk of long COVID by preventing infection altogether or by modifying the course of the infection in a way that decreases the risk of long-term sequelae. A meta-analysis of early studies of this topic before emergence of the Omicron variant concluded that there is a possible protective effect, but the available data are highly heterogeneous further complicated by the inconsistent diagnostic criteria [20]. Several more recent publications showed protective effects in adults [21-23] and in children [24,25].

Here, we used whole-population data from Czechia to estimate the time-dependent waning of post-vaccination, post-infection or hybrid immunity against SARS-CoV-2 Omicron variants in order to add Czech population data to the existing literature and try to replicate or supplement previously published studies. Furthermore, we aimed to study the possible preventative effects of vaccines against long COVID specifically in people infected with SARS-CoV-2 after their vaccination. Our source data differ from similar published studies in two important aspects: they include (i) a physician-coded indication that COVID-19 was the reason for hospitalisation, and (ii) a physician-coded diagnosis of COVID post-infection syndrome, long COVID, thus providing a different perspective compared with symptom-based estimation of this condition in other studies. Our data also include information on the type of administered vaccine, so we can discern subjects vaccinated with the updated Comirnaty Original/Omicron BA.4–5 vaccine.

## Methods

We analysed two undesirable outcomes related to SARS-CoV-2 infection: (i) severe course, indicated by the need for oxygen therapy within 30 days from the positive test and a confirmation that the primary reason for hospitalisation was COVID-19 and (ii) a long COVID diagnosis following an infection no more than 183 days later.

### Vaccine rollout in Czechia

In Czechia, vaccination against COVID-19 started on 27 December 2020, initially with the mRNA-based vaccine Comirnaty (BNT162b2 mRNA, BioNTech-Pfizer), followed by Spikevax (mRNA-1273, Moderna), and the adenovirus-based vector vaccines Vaxzevria (ChAdOx1-S, AstraZeneca) and the Janssen COVID-19 vaccine (Ad26.COV2-S, Janssen-Cilag International). The vaccines were initially available only to the oldest age cohorts. Subsequently, availability was progressively extended to additional age groups. At the start of our study period in December 2021, full vaccination against SARS-CoV-2 had been universally accessible for more than 5 months to all willing recipients 12 years and older in Czechia. The administration of booster doses began on 20 September 2021. By December 2021, boosters were freely available to all individuals who had completed their vaccination course 5–6 months earlier, depending on their age. In January 2022, this interval was uniformly shortened to 5 months for everyone. The administration of second boosters commenced on 18 July 2022 and was immediately available to all from the start. We emphasise that by complete vaccination we mean two doses of vaccine (and just one for the Janssen COVID-19 vaccine) and by booster we mean the following vaccine doses. In September 2022, the updated Comirnaty Original/Omicron BA.1 and soon after that, the Comirnaty Original/BA.4–5 became available to all adults and children older than 12 years, followed by Comirnaty XBB in September 2023. A large majority (close to 85%) of vaccine doses given in Czechia were variants of Comirnaty.

Our study period for immunity-providing events was 1 December 2021 to 16 August 2023 for severe course and 1 December 2021 to 31 March 2023 for long COVID. We studied only outcomes related to infections with SARS-CoV-2 Omicron variants, identified here as either BA1/2 or BA4/5+ (BA4/5 and later variants). Infections with BA1/2 variants denote events diagnosed by allele-specific PCR or individuals who tested positive during the period from 31 January 2022 to 23 May 2022, when the BA1/2 variant was predominant in Czechia, with a prevalence above 94% according to sequencing. Infections with BA4/5+ variants denote events identified by allele-specific PCR or individuals who tested positive between 1 August 2022 and the end of the study period, when the BA4/5 variant was predominant in the Czechia, with a prevalence above 95% or when later Omicron variants including XBB and BQ.1 were circulating along with BA4/5, accounting for almost 100% of COVID-19 cases (according to SARS-CoV-2 sequence data retrieved from the Global Initiative on Sharing All Influenza Data (GISAID) via covariants.org). As dates of the outcomes for the Cox model we take the dates of diagnosis of severe outcomes or long COVID, respectively. Therefore, to include follow-up periods in the study, we have only accounted for infections occurring up until 16 July 2023 for severe outcomes, and up to 30 October 2022 for long COVID.

### Statistical methods

To compute the effectiveness of vaccination or protection after previous infection against a severe course, we used Cox regression analysis with time-varying covariates and calendar time applied to the whole population, as in Šmíd et al. [6]. We included individuals of any age. We took infections as recurring events. We categorised the source of immunity as follows: full vaccination, first and second booster vaccinations and prior infection. A positive test was deemed a re-infection if it occurred at least 61 days after the initial infection, in accordance with the methodology adopted by the National Health Information System. We refer to the combination of vaccination and a previous infection as hybrid immunity, without differentiation based on the sequence of acquisition. For each source of immunity, we split the follow-up into as much as nine 61-day time windows, i.e. we estimated immunity waning during up to 18 months; however, for some immunity sources, such as booster doses, the periods of observation were shorter because the booster doses were introduced later. Control variables were age group, sex and Deyo–Charlson comorbidity index. Details for the Cox model setting and the pair-wise comparisons of protective effects are described in the Supplement. For the analysis of long COVID, we considered all individuals older than 18 years with a confirmed Omicron infection and a known comorbidity index and a long COVID diagnosis (according to International Classification of Diseases [26]) from the date of the infection confirmation up to 183 days after this date. We then evaluated the effectiveness of vaccination or protection after previous infection using a logistic regression with the same covariates as above.

### Data

Data for the study came from the Czech National Health Information System (NHIS) and combine data from the Czech National Information System of Infectious Diseases (ISID) with the National Registry of Reimbursed Health Services (NRRHS), which contains data from health insurance companies covering almost 100% of healthcare in Czechia) and with the mortality database. These data were aligned with the Czech population register (CPR) to acquire the demographic characteristics. Data have been anonymised for this study and do not contain information that could serve to identify particular individuals.

Our dataset thus contains anonymised records of Czech citizens and permanent residents, who are registered by any healthcare insurance company and/or are recorded within the ISID database, encompassing a total of 10,350,257 records of individuals who were alive at the start of our study period. The discrepancy between the documented records and the total population is attributed to a variety of factors, the majority of which – 491,601 instances – involve the inability in aligning the NHIS records with the CPR. The reasons for this inability may encompass data errors, ambiguity in identification or administrative delays. We also excluded 5,181 NHIS records due to data inconsistencies. After the exclusion, our dataset contained 9,853,475 individuals, representing 93.7% of the population at the end of 2021. 

For each individual, the corresponding record included all their positive SARS-CoV-2 tests, records of all COVID vaccine doses including brand and date, records of hospitalisations with COVID-19 including admission and discharge dates, treatment type (stay in intensive care, mechanical ventilation, extracorporeal membrane oxygenation, including their start and end date), an indicator issued by hospital personnel of whether or not the primary reason for hospitalisation was COVID, date of death including whether COVID was a cause of death. For hospitalisations and mortality related to COVID-19, physicians followed Czech national clinical criteria and guidelines to ensure accurate documentation and reporting. This served as a validation step that aligned clinical assessments with insurance and administrative data. Each record also included demographic data (age, sex and region of residence). For infections, some of the data included information on the virus variant based on a variant identification using the definition of viral S protein mutations according to the European Centre for Disease Prevention and Control [27]. For the majority of records, we estimated the infecting variant using the dominant variant of the specific time period (denoted below as BA1/2 or BA4/5+) as data from variant-specific PCR was unavailable. A vast majority of records (all records where linkage to National Registry of Reimbursed Health Services was successful) contained a Deyo–Charlson comorbidity index and information on whether and when the individual was diagnosed with long COVID; the identification of long COVID was based on a record of ICD-10 code U09 (post-COVID condition) [26] by a physician in the record for the health insurance company according to the definition and guidelines provided by Czech Pulmonary Medicine Society [28] which outlines the diagnostic criteria for long COVID [29] based on expert consensus and international recommendations.

## Results

**Figure 1:**
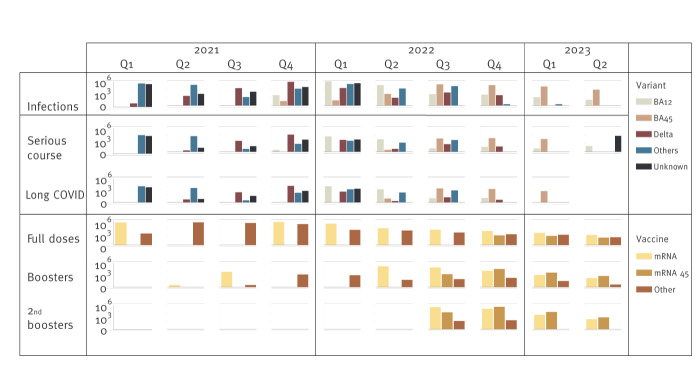
Time series of confirmed SARS-CoV-2 infections, severe disease and long COVID cases by last variant before the onset, and of vaccination status, Czechia, 2022 (n = 9,853,475)

The Table provides a demographic summary of our dataset after exclusions and a comparative analysis between it and the general Czech population, including the percentages of individuals who were infected, fully vaccinated or had received a booster by the beginning of 2022. The courses of new infections and vaccinations are displayed in Figure 1.

**Table:** Comparison of the dataset used in the present study with the official population and vaccination datasets in 2022, Czechia, 2022^a^ (n = 9,853,475)

	Population		Age (years)		Infected	Vaccination	
	Count	Ratio	Mean	Median		Full	Boost
Actual							
Male	518,3775	49.3%	41.1	42.5	Not computed^b^		
Female	533,2932	50.7%	44.0	45.1			
Total	10,516,707		42.8	43.8	23.7%	63.3%	22.9%
Dataset							
Male	482,1541	48.9%	42.3	43	Not computed^b^		
Female	503,1934	51.1%	45.3	46			
Total	9,853,475		43.8	45	23.8%	63.8%	21.9%

^a^ The official population data came from the census conducted in March 2021, and official vaccination data came from the records of the Czech Ministry of Health at beginning of 2022. From the vaccination dataset, the official percentages of infected, fully vaccinated and booster-vaccinated individuals were computed against the official population dataset (n = 10,516,707).

^b^ Comparison was not available due to lack of this information in the summary vaccination official data.

mRNA: Comirnaty or Spikevax vaccine; mRNA45: Comirnaty bivalent Original/Omicron BA.4–5 vaccine; SARS-CoV-2: severe acute respiratory syndrome coronavirus 2.

Note the logarithmic scale of the y-axes. Time is in calendar quarters.

**Figure 2:**
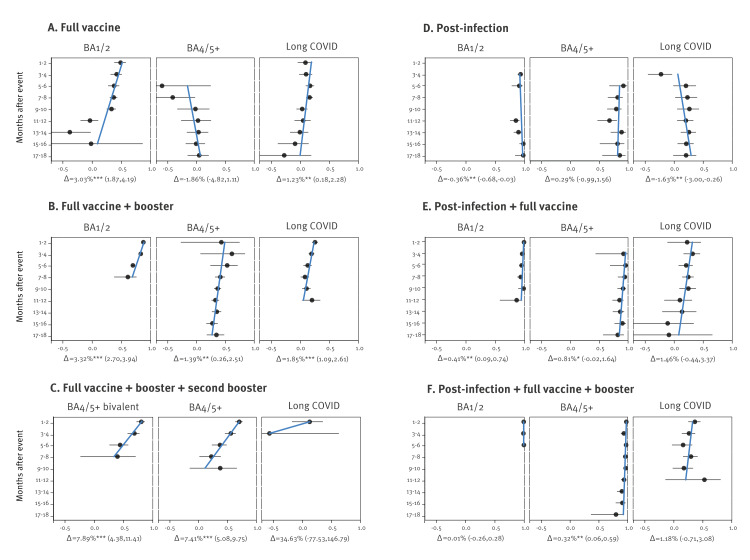
Protection by various types of immunity against a severe course of SARS-CoV-2 Omicron variants BA1/2 and BA4/5+ or long COVID, Czechia, 1 December 2021–16 August 2023 (n = 9,853,475)

CI: confidence interval; HR: hazard ratio; SARS-CoV-2: severe acute respiratory syndrome coronavirus 2.

**Figure 3:**
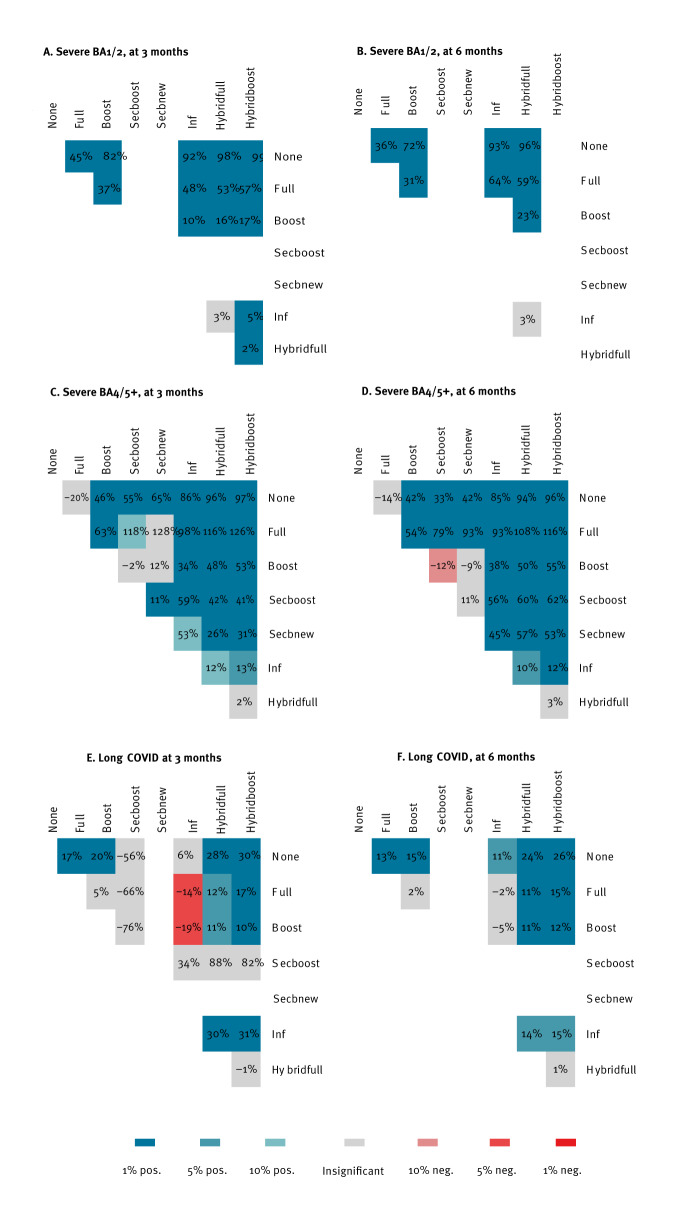
Pair-wise comparison of protective effects against a severe course of SARS-CoV-2 Omicron variants BA1/2 and BA4/5+ or long COVID at 3 and 6 months after the last immunising event, Czechia, 1 December 2021–16 August 2023 (n = 9,853,475)

HR: hazard ratio; SARS-CoV-2: severe acute respiratory syndrome coronavirus 2.

In Figure 2, we show the observed levels of protection, and the rate of weakening over time, of post-vaccination, post-infection and hybrid immunity against a severe course of COVID-19 or long COVID. In Figure 3, we show pair-wise differences in protection. All numerical outputs of our analyses are tabulated in the Supplement.

Protection is expressed as 1 − HR. Each panel shows the protective effect of immunity caused by a specific type of intervention/event (estimated at 2-month intervals), progressively more advanced vaccination series or a combination of a previous infection with vaccination (hybrid immunity), compared against a baseline of being unvaccinated and uninfected (without recorded infection or vaccination). Horizontal bars show 95% CIs of the point estimates, blue lines show linear trends over time. ∆ expresses the monthly effect change in % with a 95% CI in parentheses. The trends have been obtained by generalised least square estimation assuming a linear trend in vaccination effectiveness, significance: * 10%, ** 5%, *** 1%; see the Supplement for details and numerical results.

The numbers express the difference in protection (1 − HR) × 100% of the category in columns minus the ones in rows. The colour expresses statistical significance: red are statistically significant negative differences, green statistically significant positive differences. The categories are defined as follows: full: primary two-dose vaccine series; boost: full plus a booster dose; secboost: full plus two booster doses; secbnew: boost plus a newer, Omicron-targeting booster dose; inf: previous infection; hybridfull: inf plus a two-dose primary vaccine series; hybridboost: hybridfull plus a booster dose. The differences and their statistical significance have been obtained by comparing the generalised least square estimates of the waning trends; see the Supplement for numerical values and the methodology of comparison.

Figures 3A–B and 3C–D mostly confirm the following ordering of immunity sources by longevity and protection from the least to the most effective and stable: full vaccination < first booster < second booster < BA4/5-targeting second booster (the last two only for BA4/5+ infections) < infection ≤ hybrid full ≤ hybrid boost, where < stands for a significant difference and ≤ means less convincing difference.

### Severe course

Against a severe course of COVID-19 (BA1/2 or BA4/5+), all sources of immunity were fairly protective and persistent, see Figure 2 and the Supplement for estimated values. The only exception was the insignificant effectiveness of a full course of (original) vaccine against BA4/5+ variants: 37% (95% CI: 28–46) after 5–6 months (compared with a significant protection against BA1/2 variants after the same period). The first booster dose of the original vaccine gave better protection compared to with full vaccination, higher for the earlier variants: 70% (95% CI: 67–72) for BA1/2 and 53% (95% CI: 24–71) for BA4/5+ after 5–6 months; however, the BA4/5-targeting vaccine (2022 update) administered as a second booster mildly outperformed the second booster with the original vaccine and clearly outperformed the immunity gained from the first booster of the original vaccine. Moreover, we provide clear evidence that purely vaccine-based immunity waned over time in the protection against a severe course of disease caused by the BA1/2 or BA4/5+ viruses. This is apparent in the data concerning the introduction of boosters and their new bivalent variant: the effectiveness of full vaccination and booster dose was 88% (95% CI: 86–89) against BA1/2 after 1–2 months and decreased with 3.32% (95% CI: 2.7–3.94) per month; the effectiveness of full vaccination and second booster dose was 70% (95% CI: 64–76) against BA4/5+ after 1–2 months and decreased with 7.41% (95% CI: 5.08–9.75) per month; and the effectiveness of full vaccination and bivalent second booster dose was 80% (95% CI: 72–86) against BA4/5+  after 1–2 months and decreased with 7.89% (95% CI: 4.38–11.41) per month. In contrast, the post-infection immunity against both BA1/2 and BA4/5+ infection was fairly stable over the analysed period, with a maximum waning by only 1–2% per month.

Hybrid immunity induced by booster vaccination always outperformed both post-infection and vaccine-induced immunity. The waning was negligible for both BA1/2 and BA4/5+ variants, and the protection against severe course of COVID-19 was the best: 99% (95% CI: 96–100) against BA1/2 and 97% (95% CI: 94–99) against BA4/5+ after 5–6 months.

### Long COVID

The risk of developing long COVID after a documented infection with BA1/2 appeared to be somewhat lowered by recent booster doses or recent hybrid immunity. In case of a recent booster, the estimated effectiveness after 1–2 months was 25% (95% CI: 21–30) with a monthly waning trend of 1.85% (95% CI: 1.09–2.61); in case of recent hybrid immunity with full vaccination after 1–2 months, the estimated effectiveness was 22% (95% CI: −11 to 46) with a monthly waning trend of 1.46% (95% CI: −0.44 to 3.37). Among the analysed immunity variants, hybrid immunity with a recent booster vaccine dose showed the highest effectiveness, with 36% (95% CI: 25–46) after 1–2 months and waning at 1.18% (95% CI: −0.71 to 3.08). Older full vaccination without booster had a negligible protective effect. There may be an adverse effect of a recent previous infection before the infection connected to the long COVID diagnosis, suggesting that the risk of long COVID is increased by repeated infections.

The pair-wise comparison of immunity types in Figure 3E to some extent recapitulated the protective order of immunity sources we saw against severe cases but not for post-infection and hybrid immunity, which are less protective against long COVID suggesting, once again a deleterious effect of repeated infections. The longer-term comparison in Figure 3F, shows less pronounced differences, while also documenting the adverse effect of previous infections.

## Discussion

Using a large set of national data, we show the respective protective effects of various types of immunity against COVID-19 caused by infection with SARS-CoV-2 variants BA1/2 and BA4/5+, and against long COVID following a confirmed BA1/2 infection. Our results are broadly in agreement with previous studies of post-vaccination, post-infection and hybrid immunity against COVID in that we observed that post-vaccination immunity waned over time and that post-infection and hybrid immunity provided better protection against repeated infections than vaccine-induced immunity [1-8,11-13]. Perhaps unsurprisingly, the administration of booster doses improved the protective effectiveness of vaccination compared with the initial series, with some further beneficial effect of the updated bivalent 2022 booster.

One interesting and unexpected observation was the relatively lower effectiveness of a second booster compared with first booster at 6 months. We do currently not have a good explanation for this result, but it could be an effect of a behavioural bias related to a differential tendency to receive a second booster, as discussed below.

We also observed modest protective effects of vaccination against the development of long COVID, with more pronounced protection with recently administered booster doses and a negative effect of post-infection immunity without vaccination. Published studies on the potential protective impact of vaccination on post-infection symptoms (long COVID) have so far shown overall positive but varied results. A retrospective study conducted using Israeli healthcare data found that, compared with unvaccinated people, COVID-19-vaccinated individuals who contracted a SARS-CoV-2 infection did not have a significantly lower risk of long COVID symptoms except for prolonged dyspnoea (shortness of breath) [30]. A meta-analysis of four studies involving a total of 249,788 patients examined a range of risk factors associated with long COVID symptoms and found that those who had been vaccinated with two doses before an infection had a 40% lower risk of developing long COVID symptoms compared with unvaccinated infected individuals [31]. A systematic review concluded that a protective effect is possible but difficult to estimate due to the heterogeneity of published studies [20]. In addition, Al-Aly et al. also found that in 24 of 47 symptoms studied, the risk of long COVID symptoms was lower for vaccinated individuals compared with those unvaccinated before an infection [32]. More recent (post-Omicron) studies in adults [21-23] and in children [24,25] also reported varying protective effects of vaccination against long COVID.

In contrast to most available studies, we did not use a subset of symptoms to detect long COVID, but a physician-made diagnosis based on national guidelines [28]. Despite this different perspective, our results are in overall agreement with the published data, provide a more granular view of the various sources of immunity and pick out specifically the protective effect in individuals who had been infected with SARS-CoV-2 despite their previous immunity from the various sources.

The protective role of prior SARS-CoV-2 infections against long COVID is a complicated concept. While previous infections may confer a degree of immunity, potentially mitigating the severity or likelihood of developing long COVID, they may also contribute to its onset. This study investigated the relationship between the immune status at the time of the most recent infection before the manifestation of long COVID and it should be understood and interpreted in view of this difficulty.

Our study comes with further limitations that should be born in mind when interpreting the results. First and foremost, there are inherent limitations associated with the methodologies employed in our analysis: the Cox proportional hazards model and logistic regression. Both of these models are log-linear, presupposing the multiplicativity of risk factors (hazards for the Cox model, risks for logistic regression model). In the context of our study, this assumption implies that the hazard of experiencing a serious outcome and the risk of developing long COVID are products of the individual risk factors, including the type of immunity (and the temporal distance to its acquisition), age, sex and comorbidities. Statistically, estimating the numerous potential interactions between these factors is not feasible. Another constraint of our methods is the potential dependency of the hazards or risks on absolute time, especially if these effects vary across different groups. Another limitation may appear due to possibly different COVID-19 vaccine effectiveness with respect to age.

Similarly to other observational studies, our analysis has a limitation stemming from the fact that not all infections may be reported and recorded. Yet, this issue is less acute for severe cases. Such potential under-reporting can cause overestimation of the immunity of the unexposed population (because some individuals without a reported infection can have post-infection immunity). If the previous hidden infections occurred in the vaccinated groups, which would in turn overestimate their immunity, then these two inaccuracies could cancel each other out to some extent. However, when disparities exist in the ratio of unreported infections between compared groups, bias inevitably arises. This phenomenon may underlie the observed diminished protective effect of vaccines against the BA.4/5 variants. These data come from the time period following a large Omicron wave with potentially large numbers of unreported infections. It is plausible that individuals who had not been vaccinated were more likely to have experienced an undocumented prior infection, thereby acquiring a heightened level of post-infection immunity in comparison to their vaccinated counterparts who were shielded from infection. It should be stressed, however, that the influence of the different rate of the hidden infection on the trends of immunity waning is considerably less pronounced than its impact on the absolute values of vaccine effectiveness.

A distinctive aspect of this study is our reliance on diagnoses made by physicians (both for primary hospitalisation diagnosis and long COVID). We see this approach as more reliable than just determining these outcomes from the symptoms indirectly. The diagnoses are done according to the guidelines of the Czech Pulmonary Medicine Society, which is aligned with international standards. Although interpretation and application of these guidelines can vary among physicians and healthcare providers, our method of using physician-determined diagnostic codes ensures a robust and substantiated dataset. While we do not have the ability to verify the validity of each recorded code independently, this diagnosis-oriented approach enhances existing symptom-based research and consistently yields comparable estimates of vaccine effects.

Ultimately, findings may be influenced by biases from unmeasured confounding variables, including behavioural factors differently affecting the uptake of vaccines. For instance, in the time frame when the second booster was administered, the high protection conferred by hybrid immunity had already been known from published data. Moreover, second boosters were being recommended by experts and the media especially for high-risk individuals. Therefore, it is conceivable that individuals who received a first booster and were aware that they had also experienced but never reported a mild symptomatic case of COVID-19 during that period, considered their hybrid (unreported) immunity to be sufficient and did not opt for a second booster, while those who did may disproportionately represent the high-risk population. This effect may not be completely controlled for by the age or comorbidity index covariates in our model; consequently, a bias may occur in the group that received the second booster, resulting in the observed lower effectiveness of that second booster. Although methodologies, such as propensity score weighting or matching, exist to mitigate the effects of confounding variables, their application was not feasible in our study. The former could not be used because there is no single treatment against which the treatment propensity could be computed; instead, there are many different treatments, provided by various levels of immunity. The matching methods could not be used due to the long list of potential matching criteria, including, but not limited to, the absolute time of the treatment administration.

Finally, antiviral treatments for COVID-19 became widely available during the Omicron-dominant period (for example, Paxlovid only became available on prescription in pharmacies in the BA4/5+ period) and are preferentially given to adult patients who are at increased risk of progression to the severe form of COVID-19. As being unvaccinated, in addition to age and comorbidities, is considered an increased risk, it may bias the results for comparing types of immunity against each other. Our results show a continued importance of vaccination in the prevention of severe cases of COVID-19 but highlight the need for repeated and updated booster doses. The relevance of vaccination for the prevention of long COVID outside of the prevention of infection appears weakly supported in our analysis of the data.

## Conclusion

This study provides evidence that vaccination remains a crucial measure in preventing severe cases of COVID-19, particularly when booster doses are administered. Hybrid immunity, derived from vaccination after an infection, offers the most robust protection, with its effectiveness waning more slowly over time than vaccination alone. Despite the waning of vaccine-induced immunity, booster doses, especially those updated for Omicron variants, significantly enhance protective effects. While vaccines do provide some protection against the development of long COVID, this effect is less pronounced compared with their effectiveness in preventing severe acute cases of COVID-19. This finding underscores the need for continued vigilance and research to improve long-term protection strategies. The ongoing evolution of the virus necessitates adaptive public health strategies to sustain the benefits of vaccination and manage the disease's long-term effects.

## Supplementary Data

222000 Supplement
