# Supplementary material for: Post-vaccination, post-infection and hybrid immunity against severe cases of COVID-19 and long COVID after infection with SARS-CoV-2 Omicron subvariants, Czechia, December 2021 to August 2023
Source: Euro Surveill. 2024 Aug 29;29(35):2300690. doi: 10.2807/1560-7917.ES.2024.29.35.2300690 (PMC11484334; doi:10.2807/1560-7917.ES.2024.29.35.2300690)
Supplement: Supplement [file 23-00690_TRNKA_Supplement.pdf]

Post-vaccination, post-infection and hybrid immunity against severe cases of omicron subvariants of covid-19 and long covid: Czech Republic population study

Supplementary material

April 15, 2024

This supplementary material is hosted by Eurosurveillance as supporting information alongside the article "Post-vaccination, post-infection and hybrid immunity against severe cases of omicron subvariants of covid-19 and long covid: Czech Republic population study", on behalf of the authors, who remain responsible for the accuracy and appropriateness of the content. The same standards for ethics, copyright, attributions and permissions as for the article apply. Supplements are not edited by Eurosurveillance and the journal is not responsible for the maintenance of any links or email addresses provided therein.

In this Supplementary material, we describe the statistical methodology used for the analyses, and present a numerical results, underlying our computations and graphs.

Severe Course – Methodology and Numerical Results

For the analyses of protection provided by different sources of immunity, we use the following Cox proportional hazards model:

$$\lambda(t|\texttt{Immunity}_t, \texttt{AgeGr}, \texttt{Sex}, \texttt{DCCI}) = \lambda_0(t) \exp\{\alpha\texttt{Immunity}_t + \beta\texttt{AgeGr} + \gamma\texttt{Sex} + \delta\texttt{DCCI}\}$$

where λ is the hazard function, λ_0 is the baseline hazard function, $\texttt{Immunity}_t$ is a vector of dummy variables, describing the immunity status of an individual at t, `AgeGr` is the vector of dummy variables determining the age category, `Sex` is the dummy variable describing sex of an individual, and `DCCI` is the vector of the dummies corresponding to the following values of the Deyo-Charlson comorbidity index: $0, 1, \ldots, 5, 6+$. We take time t as absolute, i.e. in days since the study start. A clear advantage of this approach is that overall influences to the hazard like incidence, counter-epidemic measures or weather, can be captured by the baseline hazard function on which the estimates of hazard rates, used for computation of effectiveness/protection, do not depend.

The dummies for the `Immunity` covariate are coded as follows: `_noimmunity` means that the individual is unvaccinated uninfected at time t, the form `I_bbb_eee` means that the individual's current immunity is `I` and it has been acquired between `bbb` and `eee` days from t; the value `_other` stand for all the immunity statuses we do not study (partial vaccination for instance). The `I` may be: **full**, primary two-dose vaccine series; **boost**, full plus a booster dose; **secboost**, full plus two booster doses; **secbnew**, boost plus a newer, omicron-targeting booster dose; **inf**, previous infection; **hybridfull**, inf plus a two-dose primary vaccine series; **hybridboost**, hybridfull plus a booster dose. The `AgeGr` covariate indicates the age of an individual at the time of the study start and can take values $0-11$, $12-15$, $16-17$, $18-24$, $25-29$, $\ldots$, $75-79$, and 80+. The `Sex` covariate may be either `M`-male or `F`-female.

We take the confirmed infection with the subsequent need for oxygen therapy (by 30 days on) in combination with the physicians' indicator that covid was the primary reason for hospitalization, as outcome. Covid– as well as non-covid deaths are censoring events.

We take the value `_noimmunity` as reference for $\texttt{Immunity}_t$,[1] so the hazard of a non-immunized person is

$$\lambda(t|\texttt{\_noimmunity}, \texttt{AgeGr}, \texttt{Sex}, \texttt{DCCI}) = \lambda_0(t) \exp\{\beta\texttt{AgeGr} + \gamma\texttt{Sex} + \delta\texttt{DCCI}\},$$

and, consequently, the hazard ratio of the i-th immunity status is

$$h_i = \frac{\lambda(t|i, \texttt{AgeGr}, \texttt{Sex}, \texttt{DCCI})}{\lambda(t|\texttt{\_noimmunity}, \texttt{AgeGr}, \texttt{Sex}, \texttt{DCCI})} = \exp\{\alpha_i\},$$

the protection provided by the immunity i is then computed as

$$e_i = 1 - h_i.$$

Next we present the results of the Cox Regressions. Here, "lower" and "upper" stand for the bounds of 95% CI.

[1] We take age group $40-44$, male sex, and $\texttt{DCCI} = 0$ as references for `AgeGr`, `Sex`, `DCCI` respectively

	coeff	lower	upper	h	lower	upper	ϵ	upper	lower
Immunity_other	-0.19	-0.37	-0.01	0.83	0.69	0.99	0.17	0.31	0.01
Immunityboost_001-061	-2.08	-2.18	-1.98	0.12	0.11	0.14	0.88	0.89	0.86
Immunityboost_062-122	-1.77	-1.84	-1.71	0.17	0.16	0.18	0.83	0.84	0.82
Immunityboost_123-183	-1.19	-1.29	-1.10	0.30	0.28	0.33	0.70	0.72	0.67
Immunityboost_184-244	-0.94	-1.41	-0.48	0.39	0.24	0.62	0.61	0.76	0.38
Immunityfull_001-061	-0.67	-0.85	-0.48	0.51	0.43	0.62	0.49	0.57	0.38
Immunityfull_062-122	-0.54	-0.70	-0.37	0.59	0.50	0.69	0.41	0.50	0.31
Immunityfull_123-183	-0.47	-0.62	-0.32	0.63	0.54	0.72	0.37	0.46	0.28
Immunityfull_184-244	-0.46	-0.56	-0.36	0.63	0.57	0.70	0.37	0.43	0.30
Immunityfull_245-305	-0.40	-0.51	-0.29	0.67	0.60	0.75	0.33	0.40	0.25
Immunityfull_306-366	0.04	-0.11	0.18	1.04	0.90	1.20	-0.04	0.10	-0.20
Immunityfull_367-427	0.32	0.03	0.62	1.38	1.03	1.86	-0.38	-0.03	-0.86
Immunityfull_428-488	0.02	-1.95	1.98	1.02	0.14	7.27	-0.02	0.86	-6.27
Immunityhybridboost_001-061	-4.78	-5.40	-4.16	0.01	0.00	0.02	0.99	1.00	0.98
Immunityhybridboost_062-122	-4.28	-4.80	-3.75	0.01	0.01	0.02	0.99	0.99	0.98
Immunityhybridboost_123-183	-5.23	-7.19	-3.27	0.01	0.00	0.04	0.99	1.00	0.96
Immunityhybridfull_001-061	-4.69	-5.83	-3.56	0.01	0.00	0.03	0.99	1.00	0.97
Immunityhybridfull_062-122	-3.20	-3.66	-2.73	0.04	0.03	0.06	0.96	0.97	0.94
Immunityhybridfull_123-183	-3.00	-3.59	-2.41	0.05	0.03	0.09	0.95	0.97	0.91
Immunityhybridfull_184-244	-2.73	-3.36	-2.11	0.06	0.03	0.12	0.94	0.97	0.88
Immunityhybridfull_245-305	-4.21	-6.17	-2.25	0.01	0.00	0.11	0.99	1.00	0.89
Immunityhybridfull_306-366	-2.00	-3.13	-0.87	0.14	0.04	0.42	0.86	0.96	0.58
Immunityinf_062-122	-2.70	-3.22	-2.17	0.07	0.04	0.11	0.93	0.96	0.89
Immunityinf_123-183	-2.41	-3.29	-1.53	0.09	0.04	0.22	0.91	0.96	0.78
Immunityinf_306-366	-1.91	-2.42	-1.41	0.15	0.09	0.25	0.85	0.91	0.75
Immunityinf_367-427	-2.27	-2.83	-1.70	0.10	0.06	0.18	0.90	0.94	0.82
Immunityinf_428-488	-3.86	-5.24	-2.47	0.02	0.01	0.08	0.98	0.99	0.92
Immunityinf_489-549	-3.80	-5.76	-1.84	0.02	0.00	0.16	0.98	1.00	0.84
DCCI1	0.55	0.46	0.64	1.73	1.59	1.89	-0.73	-0.59	-0.89
DCCI2	0.80	0.71	0.89	2.22	2.03	2.43	-1.22	-1.03	-1.43
DCCI3	1.07	0.98	1.16	2.90	2.65	3.18	-1.90	-1.65	-2.18
DCCI4	1.18	1.08	1.27	3.25	2.95	3.57	-2.25	-1.95	-2.57
DCCI5	1.41	1.31	1.51	4.10	3.71	4.54	-3.10	-2.71	-3.54
DCCI6+	1.44	1.35	1.54	4.24	3.87	4.64	-3.24	-2.87	-3.64
DCCInodcci	1.43	1.24	1.63	4.18	3.44	5.08	-3.18	-2.44	-4.08
AgeGr0-11	-1.00	-1.39	-0.61	0.37	0.25	0.54	0.63	0.75	0.46
AgeGr12-15	-1.98	-2.82	-1.14	0.14	0.06	0.32	0.86	0.94	0.68
AgeGr16-17	-1.48	-2.49	-0.47	0.23	0.08	0.63	0.77	0.92	0.37
AgeGr18-24	-0.62	-1.05	-0.20	0.54	0.35	0.82	0.46	0.65	0.18
AgeGr25-29	-0.40	-0.83	0.02	0.67	0.44	1.02	0.33	0.56	-0.02
AgeGr30-34	-0.62	-1.05	-0.19	0.54	0.35	0.82	0.46	0.65	0.18
AgeGr35-39	-0.24	-0.62	0.15	0.79	0.54	1.16	0.21	0.46	-0.16
AgeGr45-49	0.50	0.19	0.81	1.65	1.21	2.25	-0.65	-0.21	-1.25
AgeGr50-54	0.91	0.61	1.21	2.49	1.85	3.37	-1.49	-0.85	-2.37
AgeGr55-59	1.44	1.16	1.72	4.22	3.19	5.60	-3.22	-2.19	-4.60
AgeGr60-64	1.89	1.62	2.17	6.65	5.06	8.74	-5.65	-4.06	-7.74
AgeGr65-69	2.38	2.12	2.64	10.81	8.30	14.07	-9.81	-7.30	-13.07
AgeGr70-74	2.85	2.59	3.11	17.36	13.39	22.52	-16.36	-12.39	-21.52
AgeGr75-79	3.19	2.93	3.45	24.29	18.74	31.50	-23.29	-17.74	-30.50
AgeGr80+	3.57	3.31	3.82	35.34	27.31	45.74	-34.34	-26.31	-44.74
SexM	0.40	0.35	0.44	1.49	1.42	1.56	-0.49	-0.42	-0.56

Severe Course of BA1/2

	coeff	lower	upper	h	lower	upper	ϵ	upper	lower
Immunity_other	-0.37	-0.50	-0.23	0.69	0.60	0.79	0.31	0.40	0.21
Immunitybnew_001-061	-1.09	-3.05	0.87	0.34	0.05	2.39	0.66	0.95	-1.39
Immunitybnew_062-122	-0.12	-1.51	1.26	0.88	0.22	3.54	0.12	0.78	-2.54
Immunityboost_001-061	-0.56	-1.36	0.24	0.57	0.26	1.27	0.43	0.74	-0.27
Immunityboost_062-122	-0.95	-1.83	-0.07	0.39	0.16	0.93	0.61	0.84	0.07
Immunityboost_123-183	-0.76	-1.24	-0.28	0.47	0.29	0.76	0.53	0.71	0.24
Immunityboost_184-244	-0.53	-0.67	-0.39	0.59	0.51	0.68	0.41	0.49	0.32
Immunityboost_245-305	-0.46	-0.55	-0.37	0.63	0.58	0.69	0.37	0.42	0.31
Immunityboost_306-366	-0.40	-0.49	-0.30	0.67	0.61	0.74	0.33	0.39	0.26
Immunityboost_367-427	-0.43	-0.55	-0.31	0.65	0.58	0.73	0.35	0.42	0.27
Immunityboost_428-488	-0.32	-0.45	-0.19	0.73	0.63	0.83	0.27	0.37	0.17
Immunityboost_489-549	-0.42	-0.65	-0.20	0.66	0.52	0.82	0.34	0.48	0.18
Immunityfull_123-183	0.47	-0.27	1.21	1.60	0.76	3.37	-0.60	0.24	-2.37
Immunityfull_184-244	0.35	0.03	0.67	1.42	1.03	1.95	-0.42	-0.03	-0.95
Immunityfull_245-305	0.02	-0.25	0.29	1.02	0.78	1.33	-0.02	0.22	-0.33
Immunityfull_306-366	-0.02	-0.28	0.24	0.98	0.75	1.27	0.02	0.25	-0.27
Immunityfull_367-427	-0.03	-0.22	0.15	0.97	0.80	1.17	0.03	0.20	-0.17
Immunityfull_428-488	0.01	-0.16	0.17	1.01	0.86	1.19	-0.01	0.14	-0.19
Immunityfull_489-549	-0.05	-0.23	0.14	0.96	0.79	1.15	0.04	0.21	-0.15
Immunityhybridboost_001-061	-3.74	-4.54	-2.94	0.02	0.01	0.05	0.98	0.99	0.95
Immunityhybridboost_062-122	-2.71	-3.26	-2.16	0.07	0.04	0.11	0.93	0.96	0.89
Immunityhybridboost_123-183	-3.57	-4.37	-2.77	0.03	0.01	0.06	0.97	0.99	0.94
Immunityhybridboost_184-244	-3.19	-3.79	-2.60	0.04	0.02	0.07	0.96	0.98	0.93
Immunityhybridboost_245-305	-3.34	-3.97	-2.72	0.04	0.02	0.07	0.96	0.98	0.93
Immunityhybridboost_306-366	-2.77	-3.36	-2.18	0.06	0.03	0.11	0.94	0.97	0.89
Immunityhybridboost_367-427	-2.29	-2.89	-1.70	0.10	0.06	0.18	0.90	0.94	0.82
Immunityhybridboost_428-488	-2.39	-3.19	-1.58	0.09	0.04	0.21	0.91	0.96	0.79
Immunityhybridboost_489-549	-1.59	-2.73	-0.45	0.20	0.07	0.64	0.80	0.93	0.36
Immunityhybridfull_062-122	-2.54	-4.50	-0.58	0.08	0.01	0.56	0.92	0.99	0.44
Immunityhybridfull_123-183	-3.13	-5.09	-1.17	0.04	0.01	0.31	0.96	0.99	0.69
Immunityhybridfull_184-244	-2.88	-4.02	-1.75	0.06	0.02	0.17	0.94	0.98	0.83
Immunityhybridfull_245-305	-2.45	-3.19	-1.71	0.09	0.04	0.18	0.91	0.96	0.82
Immunityhybridfull_306-366	-1.91	-2.51	-1.32	0.15	0.08	0.27	0.85	0.92	0.73
Immunityhybridfull_367-427	-1.99	-2.65	-1.34	0.14	0.07	0.26	0.86	0.93	0.74
Immunityhybridfull_428-488	-2.33	-3.21	-1.45	0.10	0.04	0.23	0.90	0.96	0.77
Immunityhybridfull_489-549	-1.72	-2.60	-0.84	0.18	0.07	0.43	0.82	0.93	0.57
Immunityinf_123-183	-2.52	-3.91	-1.13	0.08	0.02	0.32	0.92	0.98	0.68
Immunityinf_184-244	-1.71	-2.37	-1.05	0.18	0.09	0.35	0.82	0.91	0.65
Immunityinf_245-305	-1.59	-2.16	-1.02	0.20	0.12	0.36	0.80	0.88	0.64
Immunityinf_306-366	-1.13	-1.63	-0.64	0.32	0.20	0.53	0.68	0.80	0.47
Immunityinf_367-427	-2.19	-3.17	-1.21	0.11	0.04	0.30	0.89	0.96	0.70
Immunityinf_428-488	-1.70	-2.69	-0.72	0.18	0.07	0.49	0.82	0.93	0.51
Immunityinf_489-549	-1.94	-3.08	-0.81	0.14	0.05	0.45	0.86	0.95	0.55
Immunitysecbnew_001-061	-1.63	-1.97	-1.28	0.20	0.14	0.28	0.80	0.86	0.72
Immunitysecbnew_062-122	-1.16	-1.47	-0.84	0.31	0.23	0.43	0.69	0.77	0.57
Immunitysecbnew_123-183	-0.58	-0.86	-0.30	0.56	0.42	0.74	0.44	0.58	0.26
Immunitysecbnew_184-244	-0.50	-1.22	0.21	0.60	0.30	1.23	0.40	0.70	-0.23
Immunitysecboost_001-061	-1.22	-1.42	-1.01	0.30	0.24	0.36	0.70	0.76	0.64
Immunitysecboost_062-122	-0.82	-1.02	-0.61	0.44	0.36	0.54	0.56	0.64	0.46
Immunitysecboost_123-183	-0.46	-0.66	-0.26	0.63	0.52	0.77	0.37	0.48	0.23
Immunitysecboost_184-244	-0.25	-0.48	-0.01	0.78	0.62	0.99	0.22	0.38	0.01
Immunitysecboost_245-305	-0.47	-1.08	0.14	0.63	0.34	1.15	0.37	0.66	-0.15
DCCI1	0.49	0.39	0.59	1.63	1.48	1.80	-0.63	-0.48	-0.80
DCCI2	0.71	0.61	0.81	2.03	1.84	2.25	-1.03	-0.84	-1.25
DCCI3	1.00	0.90	1.10	2.72	2.46	3.01	-1.72	-1.46	-2.01
DCCI4	1.09	0.98	1.20	2.98	2.67	3.32	-1.98	-1.67	-2.32
DCCI5	1.28	1.16	1.39	3.58	3.19	4.02	-2.58	-2.19	-3.02
DCCI6+	1.42	1.31	1.52	4.12	3.72	4.57	-3.12	-2.72	-3.57
DCCInodcci	1.50	1.28	1.72	4.47	3.58	5.59	-3.47	-2.58	-4.59
AgeGr0-11	-0.79	-1.24	-0.33	0.46	0.29	0.72	0.54	0.71	0.28
AgeGr12-15	-3.36	-5.34	-1.38	0.03	0.00	0.25	0.97	1.00	0.75
AgeGr16-17	-2.51	-4.49	-0.53	0.08	0.01	0.59	0.92	0.99	0.41
AgeGr18-24	-1.86	-2.65	-1.06	0.16	0.07	0.35	0.84	0.93	0.65
AgeGr25-29	-1.46	-2.21	-0.71	0.23	0.11	0.49	0.77	0.89	0.51
AgeGr30-34	-1.16	-1.77	-0.54	0.31	0.17	0.58	0.69	0.83	0.42
AgeGr35-39	-0.79	-1.33	-0.26	0.45	0.27	0.77	0.55	0.73	0.23
AgeGr45-49	-0.28	-0.70	0.14	0.76	0.50	1.15	0.24	0.50	-0.15
AgeGr50-54	0.40	0.03	0.77	1.49	1.03	2.16	-0.49	-0.03	-1.16
AgeGr55-59	0.99	0.66	1.33	2.70	1.93	3.77	-1.70	-0.93	-2.77
AgeGr60-64	1.48	1.17	1.80	4.41	3.21	6.07	-3.41	-2.21	-5.07
AgeGr65-69	2.15	1.85	2.46	8.62	6.38	11.65	-7.62	-5.38	-10.65
AgeGr70-74	2.69	2.39	2.99	14.74	10.96	19.83	-13.74	-9.96	-18.83
AgeGr75-79	3.13	2.84	3.43	22.99	17.11	30.89	-21.99	-16.11	-29.89
AgeGr80+	3.73	3.44	4.02	41.70	31.10	55.91	-40.70	-30.10	-54.91
SexM	0.48	0.43	0.53	1.62	1.54	1.71	-0.62	-0.54	-0.71

Severe Course of BA4/5+

Long Covid – Methodology and Numerical Results

To evaluate the effectiveness of various sources of immunity against long covid, we use the logistic regression model

$$\frac{\mathbb{P}[\text{long\_covid}|\texttt{Immunity}, \texttt{AgeGr}, \texttt{Sex}, \texttt{DCCI}]}{1-\mathbb{P}[\text{long\_covid}|\texttt{Immunity}, \texttt{AgeGr}, \texttt{Sex}, \texttt{DCCI}]} = \exp\left\{-\alpha - \beta\texttt{Immunity}_t - \gamma\texttt{AgeGr} - \delta\texttt{Sex} - \eta\texttt{DCCI}\right\},$$

where the covariates have the same meaning as above. Here, we approximate the effectiveness by the odds ratio, so, for immunity i, the effectiveness is

$$\epsilon_i \doteq o_i = \exp\{-\beta_i\}.$$

Next we present the results of the regression we further use in our analysis:

	Estimate	Std. Error	z value	Pr(>|z|)
(Intercept)	-4.6345	0.0331	-140.19	0.0000
Immunity_other	-0.0682	0.0780	-0.88	0.3813
Immunityboost_001-061	-0.2923	0.0319	-9.16	0.0000
Immunityboost_062-122	-0.2145	0.0292	-7.35	0.0000
Immunityboost_123-183	-0.1360	0.0402	-3.39	0.0007
Immunityboost_184-244	-0.0840	0.0385	-2.18	0.0291
Immunityboost_245-305	-0.1129	0.0401	-2.82	0.0048
Immunityboost_306-366	-0.2247	0.0979	-2.29	0.0217
Immunityfull_001-061	-0.0930	0.0685	-1.36	0.1747
Immunityfull_062-122	-0.1043	0.0618	-1.69	0.0915
Immunityfull_123-183	-0.1839	0.0423	-4.35	0.0000
Immunityfull_184-244	-0.1745	0.0333	-5.24	0.0000
Immunityfull_245-305	-0.0328	0.0485	-0.68	0.4991
Immunityfull_306-366	-0.0497	0.0730	-0.68	0.4965
Immunityfull_367-427	0.0077	0.0779	0.10	0.9216
Immunityfull_428-488	0.0870	0.1220	0.71	0.4759
Immunityfull_489-549	0.2451	0.2292	1.07	0.2848
Immunityhybridboost_001-061	-0.4493	0.0813	-5.53	0.0000
Immunityhybridboost_062-122	-0.3029	0.0770	-3.93	0.0001
Immunityhybridboost_123-183	-0.1741	0.1037	-1.68	0.0931
Immunityhybridboost_184-244	-0.3500	0.0887	-3.95	0.0001
Immunityhybridboost_245-305	-0.1908	0.1060	-1.80	0.0719
Immunityhybridboost_306-366	-0.7499	0.4511	-1.66	0.0964
Immunityhybridfull_001-061	-0.2532	0.1846	-1.37	0.1702
Immunityhybridfull_062-122	-0.3827	0.1042	-3.67	0.0002
Immunityhybridfull_123-183	-0.2319	0.0767	-3.02	0.0025
Immunityhybridfull_184-244	-0.2785	0.0651	-4.28	0.0000
Immunityhybridfull_245-305	-0.2804	0.0941	-2.98	0.0029
Immunityhybridfull_306-366	-0.1037	0.1326	-0.78	0.4342
Immunityhybridfull_367-427	-0.1456	0.1691	-0.86	0.3892
Immunityhybridfull_428-488	0.1067	0.2622	0.41	0.6839
Immunityhybridfull_489-549	0.0871	0.5860	0.15	0.8818
Immunityinf_062-122	0.2049	0.0849	2.41	0.0159
Immunityinf_123-183	-0.2269	0.1225	-1.85	0.0640
Immunityinf_184-244	-0.2596	0.1225	-2.12	0.0340
Immunityinf_245-305	-0.3044	0.1271	-2.40	0.0166
Immunityinf_306-366	-0.2262	0.0872	-2.59	0.0095
Immunityinf_367-427	-0.2943	0.0875	-3.36	0.0008
Immunityinf_428-488	-0.2335	0.0876	-2.67	0.0077
Immunityinf_489-549	-0.2296	0.1244	-1.85	0.0650
Immunitysecboost_001-061	-0.1327	0.1513	-0.88	0.3805
Immunitysecboost_062-122	0.4501	0.7252	0.62	0.5349
DCCI1	0.5195	0.0212	24.51	0.0000
DCCI2	0.6611	0.0260	25.48	0.0000
DCCI3	0.7873	0.0316	24.91	0.0000
DCCI4	0.8746	0.0390	22.45	0.0000
DCCI5	0.9261	0.0488	18.98	0.0000
DCCI6+	1.0152	0.0409	24.85	0.0000
AgeGr18-24	-0.6757	0.0551	-12.25	0.0000
AgeGr25-29	-0.4820	0.0492	-9.80	0.0000
AgeGr30-34	-0.3398	0.0441	-7.71	0.0000
AgeGr35-39	-0.1718	0.0414	-4.15	0.0000
AgeGr45-49	0.2071	0.0359	5.77	0.0000
AgeGr50-54	0.3327	0.0371	8.96	0.0000
AgeGr55-59	0.4629	0.0366	12.64	0.0000
AgeGr60-64	0.4888	0.0398	12.28	0.0000
AgeGr65-69	0.5756	0.0404	14.26	0.0000
AgeGr70-74	0.6362	0.0415	15.34	0.0000
AgeGr75-79	0.6872	0.0445	15.45	0.0000
AgeGr80+	0.7296	0.0437	16.69	0.0000
SexM	-0.1908	0.0166	-11.48	0.0000

Figure 2 – Methodology and Numerial Results

Computation of Trend

Let $h_{t_1}, \dots, h_{t_n}$ be a series of HR's corresponding to a certain source of immunity, where the index stands for the time since obtaining the immunity. Asymptotically, by Delta Theorem[2] and Continuous mapping Theorem,

$$\mathrm{var}(h) \doteq W := T'V^{\star}T, \qquad T = \mathrm{diag}(\exp\{b_{(1)}\}, \dots, \exp\{b_{(n)}\})$$

where $(b_{(1)}, \dots, b_{(n)})'$ is the vector of b's corresponding to h and $V^{\star}$ is the corresponding sub-matrix of V.

We assume a linear trend in h, i.e.

$$h_{t_i} = \eta + \Delta t_i + \epsilon_i, \qquad i = 1, \dots, n, \qquad \mathrm{var}(\epsilon) \doteq W.$$

The GLS estimate of (η, Δ)' is given by

$$\begin{bmatrix} v \\ d \end{bmatrix} = (X'W^{-1}X)^{-1}X'W^{-1}h, \qquad X = \begin{bmatrix} 1 & t_1 \\ 1 & t_2 \\ \vdots & \vdots \\ 1 & t_n \end{bmatrix}$$

having

$$\mathrm{var}\begin{bmatrix} v \\ d \end{bmatrix} = (X'W^{-1}X)^{-1};$$

the estimate of the trend of HR's is then

$$\tau(t) = v + dt, \qquad t \geq 0,$$

and the corresponding trend of the effectiveness/protection is

$$\epsilon(t) = 1 - \tau(t) = 1 - v - dt, \qquad t \geq 0. \tag{1}$$

Numerical Results

The following tables show values, underlying Figure 2. Here, "$xxx - yyy$" stands for the time interval from xxx to yyy days after immunity acquisition, "$e(e_L - e_R)$" stands for the corresponding value of the effectiveness and its 95% CI, and "$\epsilon = z$" stands for $\epsilon(t) = z$ where t is the middle of the corresponding time interval (see Eq. (1)).

Full vaccine

	BA1/2	BA4/5+	Long covid
Δ	3.03% (1.87%-4.19%)	-1.86% (-4.82%-1.11%)	1.23% (0.18%-2.28%)
001-061	0.49 (0.38-0.57 ϵ=0.51)		0.09 (-0.04-0.2 ϵ=0.19)
062-122	0.42 (0.32-0.5 ϵ=0.45)		0.1 (-0.02-0.2 ϵ=0.17)
123-183	0.37 (0.28-0.46 ϵ=0.39)	-0.6 (-2.37-0.24 ϵ=-0.16)	0.17 (0.1-0.23 ϵ=0.14)
184-244	0.37 (0.3-0.43 ϵ=0.33)	-0.42 (-0.95–0.03 ϵ=-0.12)	0.16 (0.1-0.21 ϵ=0.12)
245-305	0.33 (0.25-0.4 ϵ=0.27)	-0.02 (-0.33-0.22 ϵ=-0.08)	0.03 (-0.06-0.12 ϵ=0.09)
306-366	-0.04 (-0.2-0.1 ϵ=0.21)	0.02 (-0.27-0.25 ϵ=-0.05)	0.05 (-0.1-0.18 ϵ=0.07)
367-427	-0.38 (-0.86–0.03 ϵ=0.15)	0.03 (-0.17-0.2 ϵ=-0.01)	-0.01 (-0.17-0.13 ϵ=0.05)
428-488	-0.02 (-6.27-0.86 ϵ=0.09)	-0.01 (-0.19-0.14 ϵ=0.03)	-0.09 (-0.39-0.14 ϵ=0.02)
489-549		0.04 (-0.15-0.21 ϵ=0.06)	-0.28 (-1-0.18 ϵ=0)

Full vaccine + booster

	BA1/2	BA4/5+	Long covid
Δ	3.32% (2.7%-3.94%)	1.39% (0.26%-2.51%)	1.85% (1.09%-2.61%)
001-061	0.88 (0.86-0.89 ϵ=0.89)	0.43 (-0.27-0.74 ϵ=0.49)	0.25 (0.21-0.3 ϵ=0.24)
062-122	0.83 (0.82-0.84 ϵ=0.82)	0.61 (0.07-0.84 ϵ=0.46)	0.19 (0.15-0.24 ϵ=0.2)
123-183	0.7 (0.67-0.72 ϵ=0.75)	0.53 (0.24-0.71 ϵ=0.43)	0.13 (0.06-0.19 ϵ=0.16)
184-244	0.61 (0.38-0.76 ϵ=0.69)	0.41 (0.32-0.49 ϵ=0.41)	0.08 (0.01-0.15 ϵ=0.13)
245-305		0.37 (0.31-0.42 ϵ=0.38)	0.11 (0.03-0.17 ϵ=0.09)
306-366		0.33 (0.26-0.39 ϵ=0.35)	0.2 (0.03-0.34 ϵ=0.05)
367-427		0.35 (0.27-0.42 ϵ=0.32)	
428-488		0.27 (0.17-0.37 ϵ=0.3)	
489-549		0.34 (0.18-0.48 ϵ=0.27)	

[2]https://www.jepusto.com/multivariate-delta-method/)

Full vaccine + booster + second booster

	BA4/5+ bivalent	BA4/5+	Long covid
Δ	7.89% (4.38%-11.41%)	7.41% (5.08%-9.75%)	34.63% (-77.53%-146.79%)
001-061	0.8 (0.72-0.86 ϵ=0.81)	0.7 (0.64-0.76 ϵ=0.7)	0.12 (-0.18-0.35 ϵ=0.12)
062-122	0.69 (0.57-0.77 ϵ=0.65)	0.56 (0.46-0.64 ϵ=0.55)	-0.57 (-5.5-0.62 ϵ=-0.57)
123-183	0.44 (0.26-0.58 ϵ=0.49)	0.37 (0.23-0.48 ϵ=0.4)	
184-244	0.4 (-0.23-0.7 ϵ=0.34)	0.22 (0.01-0.38 ϵ=0.26)	
245-305		0.37 (-0.15-0.66 ϵ=0.11)	
306-366			
367-427			
428-488			
489-549			

Post-infection

	BA1/2	BA4/5+	Long covid
Δ	-0.36% (-0.68%--0.03%)	0.29% (-0.99%-1.56%)	-1.63% (-3%--0.26%)
001-061			
062-122	0.93 (0.89-0.96 ϵ=0.92)		-0.23 (-0.45–0.04 ϵ=0.06)
123-183	0.91 (0.78-0.96 ϵ=0.93)	0.92 (0.68-0.98 ϵ=0.85)	0.2 (-0.01-0.37 ϵ=0.1)
184-244		0.82 (0.65-0.91 ϵ=0.85)	0.23 (0.02-0.39 ϵ=0.13)
245-305		0.8 (0.64-0.88 ϵ=0.84)	0.26 (0.05-0.43 ϵ=0.16)
306-366	0.85 (0.75-0.91 ϵ=0.95)	0.68 (0.47-0.8 ϵ=0.84)	0.2 (0.05-0.33 ϵ=0.19)
367-427	0.9 (0.82-0.94 ϵ=0.95)	0.89 (0.7-0.96 ϵ=0.83)	0.25 (0.12-0.37 ϵ=0.23)
428-488	0.98 (0.92-0.99 ϵ=0.96)	0.82 (0.51-0.93 ϵ=0.82)	0.21 (0.06-0.33 ϵ=0.26)
489-549	0.98 (0.84-1 ϵ=0.97)	0.86 (0.55-0.95 ϵ=0.82)	0.21 (-0.01-0.38 ϵ=0.29)

Post-infection + full vaccine

	BA1/2	BA4/5+	Long covid
Δ	0.41% (0.09%-0.74%)	0.81% (-0.02%-1.64%)	1.46% (-0.44%-3.37%)
001-061	0.99 (0.97-1 ϵ=0.99)		0.22 (-0.11-0.46 ϵ=0.31)
062-122	0.96 (0.94-0.97 ϵ=0.98)	0.92 (0.44-0.99 ϵ=0.96)	0.32 (0.16-0.44 ϵ=0.28)
123-183	0.95 (0.91-0.97 ϵ=0.97)	0.96 (0.69-0.99 ϵ=0.95)	0.21 (0.08-0.32 ϵ=0.25)
184-244	0.94 (0.88-0.97 ϵ=0.96)	0.94 (0.83-0.98 ϵ=0.93)	0.24 (0.14-0.33 ϵ=0.22)
245-305	0.99 (0.89-1 ϵ=0.95)	0.91 (0.82-0.96 ϵ=0.91)	0.24 (0.09-0.37 ϵ=0.2)
306-366	0.86 (0.58-0.96 ϵ=0.94)	0.85 (0.73-0.92 ϵ=0.9)	0.1 (-0.17-0.3 ϵ=0.17)
367-427		0.86 (0.74-0.93 ϵ=0.88)	0.14 (-0.2-0.38 ϵ=0.14)
428-488		0.9 (0.77-0.96 ϵ=0.86)	-0.11 (-0.86-0.33 ϵ=0.11)
489-549		0.82 (0.57-0.93 ϵ=0.85)	-0.09 (-2.44-0.65 ϵ=0.08)

Post-infection + full vaccine + booster

hybridboost	BA1/2	BA4/5+	Long covid
Δ	0.01% (-0.26%-0.28%)	0.32% (0.06%-0.59%)	1.18% (-0.71%-3.08%)
001-061	0.99 (0.98-1 ϵ=0.99)	0.98 (0.95-0.99 ϵ=0.98)	0.36 (0.25-0.46 ϵ=0.32)
062-122	0.99 (0.98-0.99 ϵ=0.99)	0.93 (0.89-0.96 ϵ=0.97)	0.26 (0.14-0.36 ϵ=0.3)
123-183	0.99 (0.96-1 ϵ=0.99)	0.97 (0.94-0.99 ϵ=0.96)	0.16 (-0.03-0.31 ϵ=0.28)
184-244		0.96 (0.93-0.98 ϵ=0.96)	0.3 (0.16-0.41 ϵ=0.25)
245-305		0.96 (0.93-0.98 ϵ=0.95)	0.17 (-0.02-0.33 ϵ=0.23)
306-366		0.94 (0.89-0.97 ϵ=0.94)	0.53 (-0.14-0.8 ϵ=0.2)
367-427		0.9 (0.82-0.94 ϵ=0.94)	
428-488		0.91 (0.79-0.96 ϵ=0.93)	
489-549		0.8 (0.36-0.93 ϵ=0.92)	

Figure 3 – Methodology and Numerial Results

The Figure displays the differences of protections provided by different sources of immunity. Here we explain which way we evaluate the statistical significance of the differences, which are negative differences of HR's. Say we have vectors of HR's, say $h = (h_{t_1}, \ldots, h_{t_n})'$ and another vector of HR's, say $k = (k_{t_1}, \ldots, k_{t_n})'$ assuming both to follow each their own linear trend, which implies that

$$h_{t_i} - k_{t_i} = \sigma + \alpha t_i + e_i, \qquad i = 1, \ldots, n, \qquad \text{var}(e) = U := SV^{\star\star}S',$$

$$S = \begin{bmatrix} \exp\{b_{[1]}\} & \cdots & 0 & -\exp\{b_{[n+1]}\} & \cdots & 0 \\ \vdots & \ddots & \vdots & \vdots & \ddots & \vdots \\ 0 & \cdots & \exp\{b_{[n]}\} & 0 & \cdots & -\exp\{b_{[2n]}\} \end{bmatrix}$$

where $(b_{[1]}, \ldots, b_{[2n]})'$ is the vector of b's corresponding to $(h, k)'$ and $V^{\star\star}$ is a corresponding sub-matrix of V.

The GLS estimator of $(\sigma, \alpha)'$ is

$$(s,a)' = (Z'U^{-1}Z)^{-1}Z'U^{-1}(h-k), \qquad Z = \begin{bmatrix} 1 & t_1 \\ \vdots & \vdots \\ 1 & t_n \end{bmatrix},$$

having the variance

$$M := \mathrm{var}([s,a]') = (Z'U^{-1}Z)^{-1}$$

Thus, being interested in the difference in effectiveness at a specific time t, we can estimate it as

$$\Delta_t = -s - ta,$$

having $\mathrm{var}(\Delta_t) = [1,t]M[1,t]'$

The corresponding Z-score, which is asymptotically standardized normal, so the significance can be easily determined from it, is

$$z_t = \frac{\Delta_t}{\sqrt{\mathrm{var}(\Delta_t)}}.$$

The following tables list the values of z_t, corresponding to Fig. 3

1	2	3	4	5	6	7	8
	16.58	161.74			55.24	231.64	424.58
		13.29			11.01	18.95	19.36
	-13.29				5.35	19.72	30.66
	-11.01	-5.35				1.47	2.87
	-18.95	-19.72			-1.47		3.70
	-19.36	-30.66			-2.87	-3.70	

a) severe BA1/2, at 3 months

1	2	3	4	5	6	7	8
	17.64	57.12			72.62	137.09	
		6.51			17.39	27.10	
	-6.51					11.79	
	-17.39					0.92	
	-27.10	-11.79			-0.92		

b) severe BA1/2, at 6 months

1	2	3	4	5	6	7	8
	-1.15	9.42	21.52	20.30	16.36	30.14	155.51
		3.56	1.80	1.01	5.09	6.33	6.76
	-3.56		-0.19	1.08	4.18	7.85	10.06
	-1.80	0.19		2.75	3.69	5.34	15.59
	-1.01	-1.08	-2.75		1.78	2.88	9.29
	-5.09	-4.18	-3.69	-1.78		1.88	2.38
	-6.33	-7.85	-5.34	-2.88	-1.88		0.68
	-6.76	-10.06	-15.59	-9.29	-2.38	-0.68	

c) severe BA4/5+, at 3 months

1	2	3	4	5	6	7	8
	-1.09	12.25	6.61	5.49	23.19	43.25	188.08
		4.19	2.67	2.65	6.59	7.94	8.45
	-4.19		-1.70	-0.70	6.84	11.95	15.42
	-2.67	1.70		1.25	8.32	10.59	12.27
	-2.65	0.70	-1.25		3.63	5.34	6.81
	-6.59	-6.84	-8.32	-3.63		2.27	3.18
	-7.94	-11.95	-10.59	-5.34	-2.27		1.24
	-8.45	-15.42	-12.27	-6.81	-3.18	-1.24	

d) severe BA4/5+, at 6 months

1	2	3	4	5	6	7	8
	5.85	10.86	-0.50		0.98	6.15	9.12
		1.51	-0.59		-1.97	2.28	3.98
	-1.51		-0.67		-2.31	2.09	3.06
	0.59	0.67			0.30	0.78	0.73
	1.97	2.31	-0.30			3.54	3.17
	-2.28	-2.09	-0.78		-3.54		-0.15
	-3.98	-3.06	-0.73		-3.17	0.15	

e) long covid, at 3 months

1	2	3	4	5	6	7	8
	6.07	6.93			2.35	7.70	7.46
		0.73			-0.31	3.46	3.98
	-0.73				-0.99	3.35	3.47
	0.31	0.99				2.58	2.55
	-3.46	-3.35			-2.58		0.26
	-3.98	-3.47			-2.55	-0.26	

f) long covid, at 6 months

Legand: 1 – none, 2 – full, 3 – boost, 4 – secboost, 5 – secbnew, 6 – inf, 7 – hybridfull, 8 – hybridboost.
